# Rating communication skills in dental practice: the impact of different sociodemographic factors

**DOI:** 10.1186/s12909-023-04958-y

**Published:** 2023-12-12

**Authors:** Nesreen A. Salim, Malik Sallam, Ra’ed Hisham Aldweik, Faleh A. Sawair, Aseel M. Sharaireh, Aref Alabed

**Affiliations:** 1 Prosthodontic department, School of Dentistry, Consultant in fixed and removable prosthodontics, The University of Jordan, The University of Jordan Hospital, Amman, Jordan; 2 Department of Pathology, Microbiology and Forensic Medicine, School of Medicine, The University of Jordan, Amman, 11942 Jordan; 3 Department of Clinical Laboratories and Forensic Medicine, Jordan University Hospital, Amman, 11942 Jordan; 4 Oral and Maxillofacial Surgery Resident, Jordan University Hospital, Amman, Jordan; 5 Department of Oral and Maxillofacial Surgery, Oral Medicine and Periodontology, School of Dentistry, The University of Jordan, Jordan University Hospital, Amman, Jordan; 6 Conservative department, School of Dentistry, The University Jordan, Amman, Jordan; 7 Health administration and Management consultant, International Medical Training Academy, London, UK

**Keywords:** Health communication, Doctor-patient relation, Dentist, Knowledge

## Abstract

**Background:**

Communication abilities are essential for the successful operation of a dental business and significantly influence outcomes, compliance, and patient satisfaction.

**Aims and methods:**

The aim of our study was to evaluate the knowledge and practice of doctor-patient communication among Jordanian dentists. This evaluation was conducted through a survey based on the key components of the Calgary Cambridge Observation Guides. Additionally, the impact of several sociodemographic characteristics on communication abilities was investigated. This cross-sectional study was conducted from January to June 2022. The data collection tool was an online questionnaire developed by the researchers, consisting of three sections: self-reported demographic and professional data, the practice of doctor-patient communication, and knowledge of doctor-patient communication.

**Results:**

The study included 305 dentists, comprising 106 males and 199 females, with a mean age of 32.9 ± 9.0 years. The mean score for communication skills knowledge was 41.5, indicating a moderate level of communication skills knowledge. Female dentists demonstrated significantly higher communication scores compared to their male counterparts, and those working in the private sector scored significantly higher than those in the governmental sector or in both sectors (*P* ≤ 0.05). In general, older and more experienced dentists exhibited better communication skills. Educational level had a positive impact on certain communication skills items. 58.4% believed that communication skills can always be developed and improved through training sessions, while 48.9% reported never having attended such courses. 95.1% believed that training courses on communication skills are always necessary as part of the educational curriculum. The main obstacles that may deter dentists from considering communication skills courses were limited time (62.3%), course availability (37.7%), cost (28.2%), and perceived lack of importance (8.2%).

**Conclusion:**

Among a sample of Jordanian dentists, there appears to be a discrepancy between knowledge and self-reported practices regarding communication abilities. In certain crucial, evidence-based areas of doctor-patient communication, there are fundamental deficiencies. Considering the significant role dentists play in oral health and prevention, communication skills should be a top educational priority for them.

## Introduction

Communication in dentistry is now recognized as an essential clinical skill crucial for establishing strong patient relationships, which facilitates accurate diagnosis and optimum treatment [1–3]. Patients are increasingly requesting tailored and personalized care and treatment, making patient-doctor communication a critical component of a successful healthcare system [4]. To provide patient-centered care and treatment, it’s essential to understand not only the biological aspects of their condition but also the individual experiencing it [5]. Therefore, patient-centered communication is crucial to elicit the patient’s experiences, needs, values, and preferences [2, 3, 6].

The necessity for effective interpersonal communication skills among healthcare practitioners is widely acknowledged, and these abilities can be acquired [2, 3]. Communication skills are now integrated in many dental schools’ curriculum and considered fundamental in all branches of the medical field [7]. According to a survey conducted by the Association of American Medical Colleges, communication skills are taught in almost all U.S. medical schools [8]. However, the majority of medical school courses lack a systematic structure that enables students to develop communication skills progressively in increasing challenging situations [3, 9]. Passive learning is more common than active learning and training in many disciplines is uncommon [2, 3, 10].

In Jordanian dental school curricula, the current approach remains limited to theoretical one-time course, with student assessment primarily through multiple-choice exams. These curricula lack active, skill-building activities for students. Additionally, in Jordan, there have been studies conducted by medical and nursing schools to assess the skills of their students and doctors in this area [11–13]. However, no studies have been undertaken to evaluate the communication skills of Jordanian dentists.

Communication dynamics within a dental setting differ from those in the medical and allied health fields due to the prevalent dentist-centered and patient-passive relationships [3]. To address this, it is essential to prioritize patient-centered communication training [2, 3]. Dental students should receive more hands-on experience in active listening and patient-centered techniques, utilizing methods such as role-play, video recording, and, ultimately, direct patient interaction [3, 10], especially in the case of children who may struggle to articulate their sensations or respond to questions accurately when describing their pain or symptoms, so effective communication should be developed with parents and children by building rapport and trust from the very first visit [14, 15]. Given that conventional medical consultation guidelines often prove impractical in dental contexts, a more concise guide should be implemented [3]. Furthermore, it is crucial to emphasize that communication skills should be taught and acquired with the same level of rigor as other fundamental dental skills throughout the entire dental curriculum [3, 10].

With the worldwide increase of dentists numbers, particularly in Jordan, and the significant economic impacts of COVID-19 [16], it is essential for every dentist to possess and practice great communication skills professionally in treating patients [17]. Effective interpersonal communication in dentistry has been shown to boost patient satisfaction [18, 19] and compliance [20] while decreasing patient fear [21] and the possibility of malpractice claims [22].

As the community awareness increases, and the social media role in educating the public about various treatment modalities and evidence-based dentistry [23], people seek not only skilled dentist but trustworthy one whom they can communicate with. Patients expect the dentist to greet them with a smile, listen attentively to their complains, maintain an eye contact, understand their emotions and pain, and give them uninterrupted time to speak. Dentists must also be able to persuade patients of the most suitable feasible treatment options [24].

Despite the obtainability of communication skills by clinical practice after graduation, some dentists encounter obstacles in doctor-patient communication. Additional advanced training is important to improve these skills and boost dentists’ self-esteem [7, 25, 26]. Many dentists try to overcome these obstacles by resourcing (books, articles, videos) on communication skills with the patients, or by attending professional courses led by experts in human resources and communication skills. Both approaches proved effective in enhancing dentists-patients relationship and building the trust which led to a better care and a healthier environment [24, 27, 28].

Recognizing the vital role of communication skills and their impact on overall dental competence and treatment outcomes, and considering the limited research on this topic in dental practice, this cross-sectional study aimed to assess the communication proficiency of Jordanian dentists during patient interactions. It also seeks to determine whether additional courses and training are necessary to enhance the quality and professionalism of patient care. Additionally, the study explores the influence of various sociodemographic factors on dentists’ communication skills.

## Methods

### Ethical approval

The research protocol was approved by the Ethical Committee of the Faculty of Dentistry of the University of Jordan (2451/2022/75) and in full accordance with the world medical declaration of Helsinki. All the participant dentists were informed regarding the objectives and aims of the questionnaire and agreed to fill the form.

### Study group and design

#### Study design

The current study which aimed to assess the communication skills of Jordanian dentists when treating their patients, employed a cross-sectional design with an electronic distribution of a survey over the period of January to June 2022. The survey instrument was based on a previously published validated tools including the in Calgary-Cambridge Guide, which is an important approach of teaching and training clinical communication skills, and the Dental Consultation Communications Checklist which is highly reliable, with internal consistency reliability (Cronbach’s alpha) = 0.987 [29–32]. The survey tool was uploaded into Google Forms in English language. Then, survey distribution was based on the snowball convenience-based approach starting from the first author and her contacts asking for further distribution of the survey link via multiple social media and instant messaging services; Facebook, Twitter, Instagram, LinkedIn, Messenger, and WhatsApp. Participation was voluntary, anonymous, and with no incentives upon participation. This study evaluated essential communication skills which were listed in Calgary-Cambridge Guide and the Dental Consultation Communications Checklist [29–32]. These skills comprised making much eye contact, smile, listen, lean forward, talk about the patient’s emotions, ask open questions, and share patients with treatment options and decision. Since open-ended questions are best for communication, asking questions is a talent that has to be developed [33]. However, Open-ended questions can cause rambling, irrelevant narratives which the doctor must deftly manage by pointing out to their patients [33]. Accordingly, the questions style in this study was modified to close-ended.

The inclusion criteria, as clearly stated in the questionnaire’s introduction before obtaining informed consent, encompassed two main conditions: (1) being a currently practicing dentist in Jordan and (2) possessing a high level of proficiency in the English language. Conversely, the exclusion criteria comprised four conditions: (1) being a dentist practicing outside of Jordan, (2) being a healthcare provider or medical student, (3) being an undergraduate dental student and (4) having a limited proficiency in the English language.

#### Survey instrument

The survey instrument was briefly divided into four sections as follows:

**First**, an introductory section with a mandatory e-consent item.

**Second**, sociodemographic data including six variables: age, gender, level of education, years of experience, place of work and the country of graduation.

**Third**, dentist communication section consists of 25 questions divided into 3 groups the first one (4 questions) focuses on how do dentists welcome their patients, the second one (17 questions) concentrates on how they communicate with the patient and discuss their treatment plan, the third one (4 questions) focuses on how they discuss the financial consequences of the treatment and convince the patient of it, all these questions intended to determine the level of communication of Jordanian dentists. The communication score (CS) was calculated by adding the scores for the 25 questions shown in Table 1 If the answer to each of the questions was “always” the answer was given a score of 4, “often” 3, “sometimes” 2, “rarely” 1, and “never” 0. The total CS, therefore, ranged from 0 to 100; the higher the total score the better the degree of communication of the dentists with their patients. Which were categorized by percentage based on summed scores: ≤60% represented poor knowledge,>60–80% moderate knowledge, and > 80% a good level of knowledge.

**Table 1:** The sociodemographic characteristic

Variable		Number (%)
Gender	Male	106 (34.8)
	Female	199 (65.2)
Age	Mean ± SD	32.9 ± 9.0
	Median	29
	Range	22–60
Education	Intern	22 (7.2)
	Postgraduate student/ resident	42 (13.8)
	General Practitioner	153 (50.2)
	Specialist	88 (28.9)
Experience (years)	Mean ± SD	8.7 ± 8.7
	Median	4
	Range	0–35
Work sector	Governmental	92 (30.2)
	Private	176 (57.7)
	both	37 (12.1)
Country of graduation	Jordan	229 (75.1)
	Arab countries	40 (13.1)
	Asia	4 (1.3)
	America and Western Europe	25 (8.2)
	Eastern Europe	7 (2.3)

**Fourth**, consists of six questions focuses on the necessity of communication skills courses and if the participant dentists attended any course before and if that improved their skills, moreover they were asked about the obstacles that they may face and prevent them to attend such courses, the participant dentists also asked about the kind of training they prefer, and if they have resourced (books, articles, videos) about communication skills with the patients.

Validity of the questionnaire was assessed and enhanced by three community-medicine doctors and one epidemiologist. A pre-test with 30 people was used to examine reliability. On average, 10–15 min were required to complete the knowledge and practice questions.

#### Sample size calculation

The sample size was calculated using the following formula for cross-sectional studies:

n = Z²P (1 − P)/d².

Where n = sample size, Z = 1.645 (level of confidence 90%), *P* = 0.5 (expected proportion in population) and d = 0.05 (precision).

n = (1.645)² × 0.5(1 − 0.5)/ (0.05)² (n = 270).

According to this formula, a sample size of about 270 participants is needed. For this study a sample of 305 was collected and analysed.

### Statistical analysis

Statistical analysis was performed using SPSS for Windows release 16.0 (SPSS Inc., Chicago, IL, USA). Descriptive statistics were generated and Chi-square test, Fisher exact test (when more than 20% of the cells had expected counts less than 5), independent sample t-test, ANOVA test, and Pearson’s correlation coefficient were used to examine associations between the different variables. The median was used as a cut-off value for continuous variables (age, years of experience). Multivariate linear (CS), and ordinal logistic regression analysis (communication skills) were conducted to found significantly independent variables. The significance level was set at *P* < 0.05.

## Results

Between January 2022 and June 2022, a total of 330 responses were collected. Out of these, 17 respondents (5.1%) declined to participate in the study, and an additional eight responses were excluded due to careless or inaccurate responses (2.4%). Consequently, the final study sample consisted of 305 respondents, accounting for 92.5% of the initial responses.

### Study sample characteristics

The final study sample consisted of 305 respondents, their sociodemographic characteristics are shown in Table 2. The 25 items used in assessment of communication skills (CS) and the answers of the participants are shown in Table 1.

**Table 2:** The 25 items used in assessment of communication skills and the answers of the participants

Communication skill	Always %	Often %	Sometimes %	Rarely %	Never %
1. I greet and welcome the patient as soon as they enter the clinic.	74.8	20.7	3.9	0.3	0.3
2. I make a warm smile when the patient comes into the clinic.	66.6	26.2	5.9	1.3	0
3. I leave whatever makes me busy in my hands when the patient come in the clinic.	35.7	43.3	15.4	4.3	1.3
4. I make eye contact with the patients whenever I talk to them.	74.1	22.3	3.0	0.3	0.3
5. I discuss the treatment plan thoroughly and provide the treatment options to my patients.	84.3	12.8	2.6	0	0.3
6. My decision to communicate with the patient is not based on their appearance and behavior.	28.2	24.3	21.6	17.7	8.2
7. I do not start thinking about my next question when listening to the patient’s answers.	30.8	32.1	25.2	9.2	2.6
8. I do not interrupt the patient if I disagree with a statement they have made.	26.6	28.5	29.5	13.4	2.0
9. I do not lose interest easily in conversations because most patients have nothing interesting to say.	45.2	24.9	20.3	8.2	1.3
10. I do not interrupt my patient when I have a contribution to make regarding the ongoing discussion.	18.0	33.4	29.2	17.7	1.6
11. I do not finish patient’s sentences for them when they pause and I know what they are going to say.	19.3	25.6	28.9	22.0	4.3
12. Many patients do not call me back to clarify my advice and recommendations or tell me in the next visit that they didn’t understand my advices.	24.9	42.6	19.7	10.5	2.3
13. I do not tend to say what I think, without worrying about how the patient perceives what I am saying.	36.4	26.6	21.0	13.1	3.0
14. I do not assume that I understand patients’ feelings and emotions without telling them.	11.5	15.4	32.8	31.1	9.2
15. I do not sit and listen to my patients with my arms and legs folded in front of me.	26.9	23.9	29.5	15.1	4.6
16. I do not use medical terms when discussing the diagnosis and the treatment with the patient.	16.1	32.1	32.1	16.4	3.3
17. I do not become impatient with patients who do not express their symptoms or emotions clearly.	32.1	30.2	28.2	7.5	2.0
18. I use open ended questions that cannot be answered with a simple Yes or No when talking to my patients.	5.9	32.8	30.5	19.0	11.8
19. I repeat the patient’s sentences in fewer and different words.	10.8	33.1	41.3	10.5	4.3
20. I pay attention to body language when I speak to my patients.	47.2	37.0	11.8	2.6	1.3
21. I give at least one minute to listen to my patient after the 1st question.	30.8	35.4	26.2	6.2	1.3
22. I consider myself very convincing when proposing the treatment plan to the patients (the doctors feel more pushy or forceful).	23.3	41.0	22.3	8.5	4.9
23. I feel comfortable to talk about money with the patient.	8.5	24.3	32.1	19.3	15.7
24. I find it easy to convince the patient about the cost of the treatment by sharing the benefits and outcomes of the plan.	14.4	32.8	30.8	15.1	6.9
25. I welcome the presence of accompanying person during the treatment.	30.5	27.5	27.9	8.5	5.6

#### Communication scores

The mean CS was 69 ± 9.0 (median 69, range 43–95). As shown in Table 3, female dentists had significantly higher communication scores compared with males (*P* = 0.003), and those working at the private sector had significantly higher communication scores compared with those working at governmental sector or in both sectors (*P* = 0.018). However, multivariate analysis showed that only gender was significantly associated with the CS (coefficient = 2.230, Confidence interval = 1.105 to 3.356, *P* < 0.001). CS was not affected significantly by other sociodemographic variables.

**Table 3:** Communication scores (CS) and its association with the sociodemographic variables

Variable		CS	*P* value
Gender	Male	66.9 ± 9.0	**0.003**
	Female	70.1 ± 8.8	
Age	≤ Median	69.4 ± 8.6	0.42
	> Median	68.6 ± 9.4	
	Pearson Correlation	-0.09	0.12
Education	Intern	67.0 ± 8.9	0.30
	Postgraduate student/ resident	68.1 ± 8.9	
	General Practitioner	69.9 ± 9.1	
	Specialist	68.3 ± 8.8	
Experience (years)	≤ Median	69.3 ± 8.5	0.58
	> Median	68.7 ± 9.5	
	Pearson Correlation	-0.08	0.17
Work sector	Governmental	67.6 ± 8.5	**0.018**
	Private	70.2 ± 9.0	
	Both	66.7 ± 9.1	
Country of graduation	Jordan	69.4 ± 8.9	0.16
	Arab countries	67.1 ± 10.0	
	Asia	61.5 ± 7.1	
	America and Western Europe	70.5 ± 7.4	
	Eastern Europe	66.0 ± 11.2	

#### Communication skills

In Table 4, the 25 individual skills and its association with the sociodemographic variables are seen. Female dentists practiced the communication skills more compared with male dentists and in 14 out of the 25 communication skills studied the differences were statistically significant. Age of dentists was associated with one skill; 74.5% of dentists above 29 years of age consider themselves always or often very convincing when proposing the treatment plan to the patients compared with 54.5% of younger dentists.

**Table 4:** The 25 individual communication skills and its association with the sociodemographic variables

Communication skill	*P* value					
	Gender	Age	Education	Experience (years)	Work sector	Country of graduation
1. I greet and welcome the patient as soon as they enter the clinic.	0.38	0.65	0.93	0.64	0.86	0.59
2. I make a warm smile when the patient comes into the clinic.	0.52	0.77	**< 0.001**	0.59	**< 0.001**	0.59
3. I leave whatever makes me busy in my hands when the patient come in the clinic.	0.17	0.73	0.17	0.24	0.37	0.15
4. I make eye contact with the patients whenever I talk to them.	0.37	0.065	0.79	0.10	0.90	**< 0.001**
5. I discuss the treatment plan thoroughly and provide the treatment options to my patients.	0.40	0.60	**0.01**	0.79	**< 0.001**	**< 0.001**
6. My decision to communicate with the patient is not based on their appearance and behavior.	0.17	0.30	**0.039**	0.12	0.44	0.29
7. I do not start thinking about my next question when listening to the patient’s answers.	**0.035**	0.49	0.28	0.30	0.30	0.76
8. I do not interrupt the patient if I disagree with a statement they have made.	**0.005**	0.70	0.79	0.37	0.67	0.79
9. I do not lose interest easily in conversations because most patients have nothing interesting to say.	**0.001**	0.80	**0.035**	0.98	0.13	**0.01**
10. I do not interrupt my patient when I have a contribution to make regarding the ongoing discussion.	**0.001**	0.11	0.46	0.09	0.26	0.78
11. I do not finish patient’s sentences for them when they pause and I know what they are going to say.	**0.005**	0.60	0.24	0.68	0.32	0.78
12. Many patients do not call me back to clarify my advice and recommendations or tell me in the next visit that they didn’t understand my advices.	**0.007**	0.053	0.50	0.49	0.92	0.35
13. I do not tend to say what I think, without worrying about how the patient perceives what I am saying.	**0.001**	0.07	0.20	0.23	0.56	0.71
14. I do not assume that I understand patients’ feelings and emotions without telling them.	**< 0.001**	0.85	0.10	0.90	0.35	**0.038**
15. I do not sit and listen to my patients with my arms and legs folded in front of me.	**< 0.001**	0.47	0.62	0.34	0.47	0.24
16. I do not use medical terms when discussing the diagnosis and the treatment with the patient.	**0.02**	0.71	0.79	0.64	0.60	0.50
17. I do not become impatient with patients who do not express their symptoms or emotions clearly.	**0.005**	0.66	0.13	0.86	0.64	**0.004**
18. I use open ended questions that cannot be answered with a simple Yes or No when talking to my patients.	0.27	0.17	**0.04**	**0.02**	**0.02**	0.43
19. I repeat the patient’s sentences in fewer and different words.	**0.005**	0.16	0.91	0.17	0.95	0.17
20. I pay attention to body language when I speak to my patients.	0.46	0.59	0.74	0.36	0.34	0.10
21. I give at least one minute to listen to my patient after the 1st question.	0.51	0.09	0.87	0.15	0.07	0.37
22. I consider myself very convincing when proposing the treatment plan to the patients (the doctors feel more pushy or forceful).	0.12	**0.008**	0.27	0.15	0.50	0.14
23. I feel comfortable to talk about money with the patient.	**0.004**	0.41	0.97	0.53	0.36	0.21
24. I find it easy to convince the patient about the cost of the treatment by sharing the benefits and outcomes of the plan.	**0.002**	0.12	0.62	0.40	0.12	**0.036**
25. I welcome the presence of accompanying person during the treatment.	0.31	0.32	0.16	0.56	0.65	0.33

Five communicational skills were significantly correlated to educational level. Higher percentage of specialists (90.9%), followed by general practitioners (GP) (87.4%), always or often make a warm smile when the patient comes into their clinics compared with interns (86.4%) or postgraduate students (83.3%). Higher percentage of GP (90.2%), followed by postgraduate students (83.3%), always discuss the treatment plan thoroughly and provide the treatment options to their patients compared with specialists (80.7%) or interns (59.1%). The decision to communicate with the patient was always or often based on their appearance and behavior according to 40.9% of interns, 30.8% of GP, 21.4% of postgraduate students, and 15.9% of specialists. Higher percentage of interns (18.1%), followed by specialists (10.2%) always or often lose interest easily in conversations because most patients have nothing interesting to say compared with 9.2% of GP and 4.8% of postgraduate students. Higher percentage of interns (54.5%), followed by specialists (39.8%), GP (37.3%), and postgraduate students (33.4%) always or often use open ended questions that cannot be answered with a simple Yes or No when talking to their patients.

Only one communicational skill was significantly correlated to number of years of experience, open ended questions that cannot be answered with a simple Yes or No were never or rarely used by 24.2% of dentists with four or less years of experience compared to 37.5% of dentists with more than four years of experience.

The work sector was associated with three communicational skills. Higher percentage of those working at the private sector (97.1%), followed by those working at both private and governmental sectors (91.9%), and those working at governmental sector (84.7%) always or often make a warm smile when the patient comes into their clinics. Similarly, higher percentage of those working at the private sector (91.5%), followed by those working at both private and governmental sectors (86.5%), always discuss the treatment plan thoroughly and provide the treatment options to their patients compared with those working at governmental sector (69.6%). In addition, open ended questions that cannot be answered with a simple Yes or No were always or often used by higher percentage of dentists who work at governmental sector (46.7%), followed by those working at both private and governmental sectors (45.0%), compared to those working at the private sector (34.1%).

Six communicational skills were significantly correlated to country of graduation of dentists. Graduates from Jordan (97.4%), followed by graduates from Arab countries (95%), USA and Western Europe (92%), Asia and Eastern Europe (90.9%) always or often make eye contact with the patients whenever they talk to them (*P* < 0.001). Always or often discussing the treatment plan thoroughly and providing the treatment options to their patients was the practice of graduates from Arab countries (100%), followed by graduates from Jordan (97.4%), USA and Western Europe (92%), and Asia and Eastern Europe (90.9%) (*P* < 0.001). Higher percentage of graduates from USA and Western Europe (80%), followed by graduates from Jordan (72.1%), never or rarely lose interest easily in conversations because most patients have nothing interesting to say compared with 63.7% of graduates from Asia and Eastern Europe, and 55% of graduates from Arab countries (*P* < 0.001). Graduates from Asia and Eastern Europe (63.7%) and from Arab countries (62.5%) never or rarely assume that they understand patients’ feelings and emotions without telling them more frequently than graduates from Jordan (36.3%) or USA and Western Europe (32%) (*P* < 0.001). Of the graduates from USA and Western Europe, 76% never or rarely become impatient with patients who do not express their symptoms or emotions clearly compared to 63.8% of graduates from Jordan, 54.6% of graduates from Asia and Eastern Europe, and 37.5% of graduates from Arab countries (*P* = 0.004). Graduates from Arab countries (57.5%) and from Asia and Eastern Europe (54.6%) always or often find it easy to convince the patient about the cost of the treatment by sharing the benefits and outcomes of the plan more frequently than graduates from Jordan (47.7%) or USA and Western Europe (32%) (*P* = 0.036).

When the ability to communicate effectively was notably linked with multiple sociodemographic factors, we employed multivariate analysis to examine these variables’ independent significance (Table 5). Specifically, displaying a warm smile upon a patient’s arrival at the clinic exhibited a significant association solely with the dentist’s work sector; those working in the governmental sector practice this skill less significantly compared with those working in other sectors. Conversely, none of the identified factors demonstrating a significant association with the skill of discussing treatment plans thoroughly and presenting treatment options to patients maintained their significance in the multivariate analysis. Furthermore, the ability to sustain interest in conversations, particularly in situations where patients may not have particularly engaging topics to discuss, exhibited significant associations only with gender (males less significantly than females), with no notable association with education and the country of graduation.

**Table 5:** Ordinal logistic multivariate analysis to examine the variables’ independent significance

Communication skill	Variable		Coefficient	*P* value	95% confidence interval	
I make a warm smile when the patient comes into the clinic.	Work sector	Governmental sector	-1.062	0.012	-1.896	-0.229
		Private sector	-0.029	0.946	-0.857	0.800
		Both	Reference			
I discuss the treatment plan thoroughly and provide the treatment options to my patients.	Gender	Male	-0.731	0.002	-1.184	-0.278
		Female	Reference			
I do not assume that I understand patients’ feelings and emotions without telling them.	Gender	Male	-0.894	< 0.001	-1.344	-0.444
		Female	Reference			
	Country of graduation	Jordan	0.054	0.888	-0695	0.802
		Arab countries	-1.035	0.027	-1.951	-0.118
		Asia	-0.375	0.573	-1.679	0.928
		America and Western Europe	Reference			
I do not become impatient with patients who do not express their symptoms or emotions clearly.	Gender	Male	-0.468	0.037	-0.909	-0.027
		Female	Reference			
	Country of graduation	Jordan	-0.354	0.361	-1.114	0.406
		Arab countries	-0.995	0.033	-1.912	-0.079
		Asia	-0712	0.283	-2.011	0.587
		America and Western Europe	Reference			
I find it easy to convince the patient about the cost of the treatment by sharing the benefits and outcomes of the plan.	Gender	Male	0.754	0.001	0.309	1.199
		Female	Reference			
	Country of graduation	Jordan	0.939	0.014	0.188	1.689
		Arab countries	1.444	0.002	0.531	2.357
		Asia	0.804	0.223	-0.489	2.096
		America and Western Europe	Reference			

Additionally, the skill of not presuming to comprehend patients’ emotions without their explicit expression was found to be significantly associated with both gender (males less significantly than females) and country of graduation (graduates of Arab countries less significantly compared with graduates of America and Western Europe). Similarly, the skill of avoiding impatience when patients have difficulty articulating their symptoms or emotions was found to be significantly associated with both gender (males less significantly than females) and country of graduation (graduates of Arab countries less significantly compared with graduates of America and Western Europe). None of the identified factors demonstrating a significant association with the practice of employing open-ended questions that require more than a simple “Yes” or “No” response during patient interactions maintained their significance in the multivariate analysis. Lastly, the skill to find it easy to convince the patient about the cost of the treatment by sharing the benefits and outcomes of the plan showed a significant association with gender (males more significantly than females and the country of graduation (graduates of Arab countries and Jordan more significantly compared with graduates of America and Western Europe).

#### Attitude toward training

Out of the total sample, 58.4% believe communication skills can be always, 29.8% often, 8.9% sometimes, 1.6% rarely, and 1.3% never developed and improved in training sessions; without significant association with any of the sociodemographic variables.

When asked if they have attended dentist patient communication skills course, 48.9% answered they have never, 12.5% rarely, 15.1% sometimes, 11.5% often, and 12.1% always attended such courses. Higher frequency of male dentists reported that they have always or often attended such courses (35%) compared with female dentists (17.6%) (*P* < 0.001). In addition, higher percentage of interns (31.8%), compared with specialists (26.1%), GP (24.2%), and postgraduate students (11.9%) reported that they have always or often attended such courses (*P* = 0.031). Graduates from Asia and Eastern Europe (54.5%) always or often attended the courses more frequently than graduates from Arab countries (35%), graduates from USA and Western Europe (32%) or from Jordan (19.2%) (*P* = 0.033). The courses were always or often attended more by dentists older than 29 years of age (33.6%) compared to younger dentists (14.1%) (*P* < 0.001) and by dentists with more than four years of experience (31%) compared to dentists with four or less years of experience (15.3%) (*P* = 0.008).

When the participant dentists were asked about what obstacles that may prevent them from considering training courses on communication skills, 62.3% reported that they have limited time, 37.7% that such courses were not available, 28.2% that the courses were costly, and 8.2% that it is not important. Higher percentage of interns (50%) compared to 34.6% of GP, 17% of specialists, and 16.7% of postgraduate students think that the courses are costly (*P* = 0.001). Costly was also reported by graduates from Asia and Eastern Europe (54.5%) more frequently than graduates from Arab countries (35%), graduates from Jordan (28.2%), or graduates from USA and Western Europe (4%) (*P* = 0.008), and by dentists with ≤ 4 years of experience (34.6%) compared to those with more years of experience (21.7%) (*P* = 0.012). That they have limited time to attend such courses was reported by 71.6% of specialists, 69% of postgraduate students, 57.5% of GP, and 45.5% of interns (*P* = 0.042); by 71.1% of dentists > 29 years of age compared to younger dentists (53.8%) (*P* = 0.002), and by 71.7% of dentists with > 4 years of experience compared to those with ≤ 4 years of experience (52.9%) (*P* = 0.001). That such courses are not available was reported more by dentists ≤ 29 years of age (50%) compared to older dentists (24.8%) (*P* < 0.001), and by dentists with ≤ 4 years of experience (47.7%) compared to those with more years of experience (27.6%) (*P* < 0.001).That such courses are not important was reported more by dentists ≤ 29 years of age (12.2%) compared to older dentists (4%) (*P* = 0.009), and by dentists with ≤ 4 years of experience (12.4%) compared to those with > 4 years of experience (3.9%) (*P* = 0.007).

When the participants were asked about the kind of training they prefer, 51.8% preferred comprehensive courses, 36.7% preferred single brief training, and 11.5% reported that they do not need any kind of training; without significant association with any of the sociodemographic variables.

Of the studied sample, 7.5% reported that they have always, 19.3% often, 24.9% sometimes, 20% rarely, and 28.2% never resourced (books, articles, videos) about communication skills with patients. Higher percentage of male dentists (54.7%) compared with females (33%) (*P* = 0.035), and dentists older than 29 years of age (34.2%) compared to younger dentists (19.9%) (*P* = 0.001), and dentists with > 4 years of experience (31.5%) compared to those with ≤ 4 years of experience (22.2%) (*P* = 0.013) always or often resourced (books, articles, videos) about communication skills with patients.

Of the surveyed dentists, 95.1% believe that training courses on communication skills are necessary as part of educational curriculum; this was the belief of 100% of interns, 97.7% of specialists, 95.4% of GPs, and 85.7% of postgraduate students (*P* = 0.027).

## Discussion

Knowledge and technical abilities alone do not define successful practice in the professions of dentistry and medicine [2, 3, 27]. The ability to actively listen, efficiently obtain and communicate information, manage patient emotions delicately, exhibit empathy, and be attentive are all essential components [27, 34]. The dearth of research on communication and soft skills in dentistry is a reflection of the current undervaluation of teaching in this area [2, 3].

In this study, our findings showed that while the dentists had a moderate degree of knowledge on doctor-patient communication, they reported using these skills insufficiently. In a previous study evaluating knowledge and practice of communication skills, most of family physicians had a good level of knowledge, however, there was a potential gap between knowledge and self-reported practices toward communication skills [35]. Sun et al. assessed 7 primary care providers (182 consultations), in their interviews, they reported that physicians were doing poorly and the performance of the participants was more influenced by their personalities and experiences than by their knowledge [36]. This suggests that in addition to a positive outlook, a willingness to learn, and self-efficacy, the idea of doctor-patient communication as a set of learned abilities has to be taught more effectively.

Female dentists in this study had significantly higher communication scores compared with males. Patients prefer their doctors to communicate with them in a patient-centered manner [37]. Research demonstrates unequivocally that female doctors exhibit more patient-centered communication [38]: they form more partnerships, exhibit more empathy, express more encouragement, and schedule longer appointments, to name a few examples [38]. Furthermore, when treated by a female doctor, older individuals with medical conditions who were hospitalized and receiving care from general internists had lower mortality and readmission rates [39]. More than 1.5 million hospitalizations were examined in this investigation. Therefore, it follows that patients should, on average, benefit from receiving care from a female doctor, and they should feel considerably better about their experience overall [39]. A previous article, which explored how gender preconceptions may contribute to the disparities between how patients perceive and rate the communication styles of male and female doctors, showed that findings cannot be solely attributed to the gender stereotypes [40]. Fortunately, male dentists in this study reported that they have always or often attended communication skill courses more significantly than female dentists. Although these skills depend on a variety of individual circumstances and personal factors, research has shown that training and experience can improve communication skills [41, 42].

Dentists who work at the private sector had significantly higher communication scores compared with those working at governmental sector, according to our findings. This could be attributed to the fact that there is a lot of time pressure on doctors who work in the public sector, and this problem is particularly prevalent in underdeveloped nations where people frequently wait for hours to see a doctor since they do not schedule appointments in advance. Doctors must spend less time with each patient in order to reduce waiting times, which makes it more difficult to communicate effectively [36]. A high workload was listed as one of the most important barriers to improve physician-patient communication in a primary care setting [36]. However, according to a research done in Saudi Arabia, public sector doctors inspire greater levels of patient trust than do private sector doctors. This might be because public sector doctors are more skilled in communicating with patients [43]. South Australia and Cambodia have also reported comparable findings [44, 45].

In this study, around 60% believe that communication skills can be always developed and improved in training sessions, however in this study, 50% have never attended dentist-patient communication skills courses. Communication skills (CS) may be acquired and kept with appropriate teaching and training [41, 42]. However, it is insufficient to teach only individuals who admit to having communication problems, since it turns out that those who are most confident about their communication abilities are frequently the worst at speaking with patients [46]. Using approved evaluation tools, trainers might identify people who lack communication skills and provide them instruction [47]. Programs for communication skills training enhance patient outcomes and boost doctors’ own wellbeing [48], and doctors with strong communication had less doctors’ burnouts [49]. On the other hand, several medical defense companies in the USA provide insurance price reductions to physicians who attend communication courses [50].

The level of education in this study showed no direct impact on communication skills. However, specialists showed positive and efficient communication skills in most aspects. Possible causes for failing to show effective communication in few aspects include; a weak healthcare system, a heavy workload for doctors, a lack of communication skills training in medicine, time limitation, and innate characteristics of the practitioner [36, 40]. In a previous study, there was no relation between the level of education and the communication skills for a sample consisted of interns, residents and faculty members, as all showed weak communication skills [51]. Interns were the most common category to attend communication courses (32%) in this study, however, postgraduate students’ category was the least to attended communication skills’ courses and 70% of this category reported that time limitation prevents them from considering training courses on communication skills while cost was the obstacle for interns. In a previous study, the students’ scores, after finishing the communication course, revealed that they valued communication skills as a part of their education much more than they had before [27]. Accordingly, the value of such courses cannot be appreciated unless the practitioners have taken and applied these skills [26]. Increased patient satisfaction, higher patient compliance with dental advice, less patient anxiety, and a decline in formal complaints and malpractice claims are a few advantages that have been observed when dentists exhibit strong and effective communication skills [21, 22]. Time limitation should not be an obstacle for training when considering all these benefits.

The main obstacles that prevent dentists in this study from considering training courses on communication skills were: limited time (62.3%), courses’ availability (37.7%), cost (28.2%), and that it is not important (8.2%). Limited time was obstacle for specialists, postgrad, older dentists and those with longer experience. This could be related to the high workload in these categories [36], compared to cost as an obstacle for interns, younger dentists and those with shorter experience as financial issues are more related .

Older age and more experience have a profound positive effect on attitudes and awareness regarding communication skills in this study. Dentists who are older than 29 years and those with experience more than 4 years are always or often resourced (books, articles, videos) about communication skills with patients, are always or often very convincing when proposing the treatment plan to the patients, using open questions, attending always or often communication skills courses more than younger age and lesser experience. Our findings were consistent with those of earlier studies by Hydarzade et al. and Al-Zahrani et al. [51, 52], as more professional experience and community involvement could result in improved practice.

Country of graduation of dentists showed different results for different aspects of the tested communication skills in this study. For example, graduates from Arab countries always or often make eye contact with the patients whenever they talk to them and always or often discussing the treatment plan thoroughly and providing the treatment options to their patients more than graduates from USA and Western Europe. Graduates from USA and Western Europe, never or rarely become impatient with patients who do not express their symptoms or emotions clearly compared to other graduates. Graduates from Asia and Eastern Europe always or often attended the courses more frequently than graduates from other counties, this could be related to the curriculum of the postgrad program. For example, according to a survey done by the Association of American Medical Colleges, communication skills are taught in some capacity at almost all USA medical schools [8], the standards for predoctoral accreditation in American dentistry schools now include the subjects of communication and interviewing [53].

Moreover, culture-specific expectations for patient-doctor interactions may be a factor in explaining communication scores [35]. There are intercultural disparities in how doctors communicate, according to earlier studies [35, 54]. For instance, a study by Matusitz et al. that compared the doctor-patient communication styles of American doctors with those of doctors from Pakistan, Japan, and Thailand revealed significant differences between the communicative styles of American doctors and those of Asian countries, with Asian doctors being more authoritative and conducting most of the talking. Aspects of the doctor-patient interaction include religious or philosophical perspectives on healthcare, a paternalistic approach to patients, and collectivistic and scripted communication methods were shared by doctors in Asian nations [54].

This study assessed critical communication abilities, which hold great significance as they align with the principles outlined in the Calgary-Cambridge Guide, a pivotal framework for instructing and honing clinical communication skills [32], such as: making much eye contact, smile, listen, lean forward, talk about the patient’s emotions, ask open questions, and share patients with treatment options and decision [36]. Since open-ended questions are best for communication, asking questions is a talent that has to be developed [33]. Nonetheless, it’s important to note that the use of open-ended questions can sometimes elicit lengthy and unrelated narratives from patients, which healthcare professionals must skillfully manage by redirecting the conversation [33]. When no more open questions can be asked and the patient has withdrew, closed questions may be utilized [55]. While doctors should listen to patients without interrupting for the first minute or two, many are unable to do so and begin to speak to patients just after 18 s of narration [56]. In this study only 31% give at least one minute to listen to patients after the 1st question. According to a prior study on refugees’ satisfaction with health care, patient satisfaction is influenced by a variety of individual factors, including the staff’s communication skills, their ability to listen to patients’ complaints and provide in-depth treatment explanations. These factors are not only related to expensive interventions and the development of complex medical and dental services [19].

### Strength and limitations

To the best of our knowledge, this is the first research of dentists’ understanding and practice of doctor-patient communication. The impact of several sociodemographic variables on communication abilities was examined. The sample size was appropriate, and the findings offer managers reliable data. This study has certain limitations; assessment of patients’ opinions and satisfaction about their dentists’ communication abilities were not performed, which may have an impact on the results of their treatment. The second restriction was the dearth of publications on this target demographic, which made comparisons challenging. Additionally, a limitation of this study is the absence of a validation process for the questionnaire, the survey instrument utilized in this study drew its foundation from the Calgary-Cambridge Guide and the Dental Consultation Communications Checklist (DCCC). While the CCOG is typically employed as an observational guide for clinical assessments, in this study, it was adapted for use as a survey tool. Notably, previous research in the field of communication studies, as indicated by the validating authors, has recommended the appropriateness of both CCOG and DCCC for guiding consultations. These tools have been deemed feasible for routine application as assessment instruments for dental students and practitioners, demonstrating reliability in their assessments [29–32]. So, as a limitation of this study is missing validation process of the questionnaire.

Future research should consider incorporating interviews of focused groups as they can offer valuable insights that may not be fully captured through surveys alone.

## Conclusion

It appears that having a high degree of education may not translate into effective practice since there may be a discrepancy between knowledge and self-reported behaviors regarding communication skills among a sample of dentists might exist. In certain crucial evidence-based areas of doctor-patient communication, they have basic flaws. Given that dentists play a significant role in oral health and prevention, communication skills should be included as a top educational goal for dentists and given enough weight in objective, systematic clinical assessments with timely feedback. We must equip our dentists with a positive attitude and self-efficacy in doctor-patient communication with practical applications. To determine if the novel therapies are helpful, more studies will be needed. The findings of this study may aid researchers in their efforts to comprehend communication problems in primary healthcare dental services, as well as to develop and suggest solutions to enhance doctor-patient communication and reduce doctor-patient conflicts.

## Data Availability

All collected data from patients analysed during this study are included in this published article. Some datasets are available from the corresponding author on reasonable request.

## Abbreviations

CS: Communication skills
GP: General Practitioner
SPSS: Statistical Package For The Social Sciences
USA: United States of America
